# Understanding the dignity experience of South African patients in primary palliative care

**DOI:** 10.4102/safp.v67i1.6047

**Published:** 2025-04-30

**Authors:** Raksha Balbadhur, Elizabeth Gwyther

**Affiliations:** 1 Division of Interdisciplinary Palliative Care and Medicine, Department of Family, Community and Emergency Care, Faculty of Health Sciences, University of Cape Town, Cape Town, South Africa; 2 Department of Family, Community and Emergency Medicine, Faculty of Health Sciences, University of Cape Town, Cape Town, South Africa

**Keywords:** South Africa, dignity, palliative care, cancer, pain, psycho-existential distress, compassionate care, AIDS

## Abstract

**Background:**

To meet the goals of primary palliative care for patients with advanced disease and to provide holistic patient-centred compassionate care that respects the experience of patients, dignity in its entirety needs to be understood from the patients’ perspective. There are no studies to understand the predominant factors that impact the dignity experience of South African patients with advanced disease.

**Methods:**

This was a descriptive qualitative study where a semi-structured interview guide was used to understand the dignity experience of adult patients with advanced disease (Stage IV cancer, AIDS, people living with human immunodeficiency virus [HIV]), receiving home care from two hospices in the North of Durban, KwaZulu-Natal. Purposive sampling was used to identify 14 patients from culturally, linguistically and socioeconomically diverse populations representative of South Africa. In-depth interviews allowed for an exploration of participants’ lived experiences. Interviews were audiotaped and transcribed verbatim. Data were analysed using thematic analysis.

**Results:**

Four major themes and numerous sub-themes defined the total dignity experience. The themes were: (1) Physical Concerns; (2) Psychological Concerns and coping mechanisms; (3) Social Concerns; and (4) Spiritual Concerns and coping mechanisms. Sub-themes are described in the main article.

**Conclusion:**

With awareness of the factors that affect the total dignity experience, healthcare providers can be considerate of and offer optimal dignity-conserving compassionate care to respect and improve the quality of life of South African patients living with advanced disease.

**Contribution:**

This study extensively explored new knowledge on the total dignity experience of South African patients with advanced disease.

## Introduction

This article is based on the study reported in the authors’ thesis entitled ‘*Understanding the dignity experience and exploring the impact of dignity therapy and guided imagery on patients with advanced disease – a South African perspective*’.^1^ Dignity is defined as ‘the quality or state of being worthy, honoured or respected’.^2^ In studies on human dignity the concepts of intrinsic and extrinsic dignity have been described.^3^ Intrinsic dignity is recognised as an innate, inherent, universal quality that is intrinsic to being human. In contrast, extrinsic dignity is a subjective perception, shaped by how one views oneself and how one is perceived by others; it is situational in nature. During serious illness, the loss of independence and control, the treatment received from others, and the accompanying physical and emotional suffering can lead to the perception and experience of diminished extrinsic dignity.^1^

A basic principle of holistic patient-centred palliative care is to help patients with advanced disease live and die with dignity, through good symptom control and psychosocial and spiritual support to optimise quality of life.^4,5^ Primary palliative care is palliative care provided by primary health care providers (HCPs) for people living at home in the community. Whilst explored in numerous studies in Western countries,^3,4,5,6,7,8,9,10,11^ dignity in advanced disease is an essential concept that has not been explored empirically in primary care in South Africa, a country that has a diversity of cultures.

**FIGURE 1:**
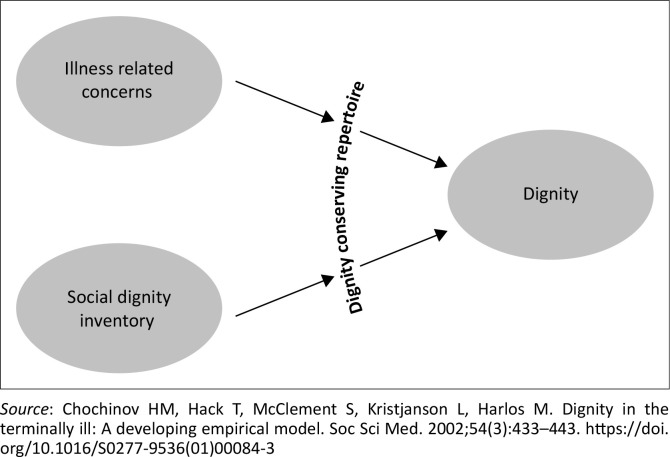
Dignity model.

To gain insight into the experience of dignity from the perspective of patients, Chochinov and his colleagues conducted a series of studies aimed at identifying the factors that either support or undermine the dignity of individuals facing life-threatening illnesses, regardless of the level of care they receive. This research culminated in the development of a dignity model,^6^ which is illustrated in Figure 1. According to this model, the loss of extrinsic dignity may result from advanced illness and a person’s social circumstances. However, approaches to enhance and preserve dignity counter this loss of extrinsic dignity.

Based on this model, the dignity interventions, Dignity Psychotherapy^12^ and the ABCD (Attitude, Behaviour, Compassion, Dialogue) of dignity-conserving care^13^ were produced to enhance dignity in patients with advanced illness. Dignity psychotherapy includes an enquiry into a patient’s history, accomplishments, meaningful roles, wishes and advice for loved ones, which is captured as a legacy document and returned to the patient.

Brennan explains that a person may possess intrinsic dignity whilst experiencing indignities, and the contrast between the two can be defined as suffering.^3^ The goal of dignity-conserving care is that the ‘loss of attributed dignity does not rob a person of intrinsic human dignity’.^3^ Compassionate care that respects the individuality of each patient and their unique experience of illness can sustain and enhance the individual’s perception of extrinsic dignity.

To uphold and support the dignity of a dying person the concept of dignity must be examined from the perspective of the patient experiencing death. The rationale for this study was that there is limited literature on the dignity experience at the end of life in the South African setting. Mnyandu, in an article exploring the concept of Ubuntu in relation to dying and dignity in palliative care, states that Ubuntu is analogous to human dignity.^14^ Ubuntu is an African worldview for those who ascribe to it^15^ and translates to ‘*umuntu ngumuntu ngabantu*’ or ‘a person is a person because of other people’. Importance is given to the ‘individual as being embedded in a community’.^16^ There is recognition of one’s innate worth and that communal relationships with others influence one’s worth. The values central to Ubuntu are group solidarity, kinship, harmony, spirituality, respect, caring, sharing, dignity and compassion for all.^17^ This qualitative research study aimed to inform the understanding of the dignity experience of patients living with advanced disease in hospice care from diverse socioeconomic and cultural perspectives. This understanding can support HCPs to offer optimal dignity-conserving care to the dying.

## Research methods and design

### Study design

This was a descriptive qualitative study.

### Setting

Participants recruited for this study conducted in 2016 were identified by hospice staff using purposive sampling. They were from diverse socioeconomic and cultural backgrounds living with advanced disease and receiving home care from Verulam Regional Hospice and Dolphin Coast Hospice in KwaZulu-Natal. KwaZulu-Natal is a province of South Africa, consisting of isiZulu-speaking black people (82%), white people (9.4%), Indian people (6.6%) and mixed race people (1.4%).^18^ Women play a central role in the family and the value of Ubuntu is important in Zulu culture.^19^ Participants belonged to high-income, middle-income, and low-income urban and peri-urban households and diverse racial and cultural backgrounds.

### Study population

Patients eligible for the study were adults with advanced disease (Stage IV cancer, people living with human immunodeficiency virus (HIV) with advanced co-morbid disease) whose first language is English or isiZulu. Patients who were too frail to take part in the study and those with cognitive impairment as measured by the Mini-Mental State Examination (MMSE) < 24 were not eligible for the study. With a heterogeneous elderly South African population, Ramlall et al. found that a cut-off score of < 24 screened positive for cognitive impairment.^20^ Amongst the 14 hospice patients interviewed, one participant was deemed ineligible for the study. Additionally, one potential participant passed away following the first interview and thus did not complete the study.

### Study tools

Two study tools were used to collect data. The first was a questionnaire designed to gather necessary demographic information of the participants. The second tool was a semi-structured interview guide (Table 1)^1^ for an in-depth interview with participants. This interview guide was developed and validated by Chochinov et al. in 2000.^6^ After consulting with colleagues and the supervising researcher involved in hospice care, we determined that the interview guide was suitable for our cultural context and required no modifications. The Chochinov model of dignity in the terminally ill^6^ (Figure 1) was used as the theoretical framework for the study to understand how dying patients define and understand dignity. The interview guide was translated into isiZulu, ensuring that it was accessible in both English and isiZulu. This bilingual approach facilitated effective communication and comprehension for participants, enhancing the validity of the research findings.

**TABLE 1:** Semi-structured interview guide with questions that explored each patient’s sense of dignity.

Question number	Question
1	In terms of your own illness experience, how do you define the term dignity?
2	What supports your sense of dignity?
3	What undermines your sense of dignity?
4	Are there specific experiences you can recall in which your dignity was compromised?
5	Are there specific experiences you can recall in which your dignity was supported?
6	What would have to happen in your life for you to feel that you no longer had a sense of dignity?
7	Some people feel that life without dignity is a life no longer worth living. How do you feel about that?
8	Do you believe that dignity is something you hold within you, and/or is it something that can be given or taken away by others?

Source: Chochinov HM, Hack T, McClement S, Kristjanson L, Harlos M. Dignity in the terminally ill: A developing empirical model. Soc Sci Med. 2002;54(3):433–443. https://doi.org/10.1016/S0277-9536(01)00084-3

### Data collection

The primary palliative care physician carried out all the interviews for this research and enlisted an isiZulu-speaking hospice caregiver. This caregiver received training in research ethics and methodology to improve cultural understanding and maintain ethical integrity throughout the research process. The isiZulu assistant provided translation in the interviews of four isiZulu-speaking patients. The researcher used the interview guide and depending on the participants’ responses, explored the areas described as impacting their dignity. Field notes were taken during all interviews. All interviews were recorded and then transcribed word-for-word. Interviews of isiZulu-speaking participants were translated during the interview by the research assistant and audiotaped for transcribing. The researcher constantly reviewed the transcripts as they were produced and familiarised herself with the data, identifying any key issues that had been omitted. These issues could be explored in successive interviews. Recruitment continued to data saturation (whereby no new themes emerged from the interviews),^21^ which was reached at 12 participants.

### Data analysis

The data were analysed manually using thematic analysis in line with recommendations by Clarke et al.^21^ following the steps of familiarisation and immersion in data, coding and induction of themes and sub-themes. General themes became evident whilst conducting the interviews and transcribing the recorded interviews. Discussion with the study supervisor assisted in refining and finalising the themes and subthemes.

### Data confidentiality

Each participant was given a unique code, which was also used to label all associated interviews, questionnaires and audio recordings for identification purposes. The hardcopy data and audiotapes for the participants were securely stored in the researcher’s locked office, whilst the Excel datasheet providing information on the codes and corresponding participants was on the researcher’s password-protected laptop.

### Ethical considerations

Ethical clearance to conduct this study was obtained from the University of Cape Town Human Research Ethics Committee on 01 December 2015. The ethical clearance number is 578/2015. All participants gave oral and written consent to partake in the study.

## Results

The demographic characteristics of participants are listed in Table 2.

**TABLE 2:** Summary of demographics of participants (*N* = 12).

Variable	*n*	%
**Gender**		
Male	7	58
Female	5	42
**Age (years)**		
31–45	4	33
46–65	3	25
66–81	5	42
Diagnosis: Cancer	10	83
Prostate	3	-
Bladder (1 with AIDS)	2	-
Anal (with AIDS)	1	-
Brain	1	-
Ovarian	1	-
Uterine (with AIDS)	1	-
Breast	1	-
Diagnosis: HIV	5	42
With cancer (above)	3	-
With comorbidities (CVA or CCF)	2	-
**Ethnicity**		
Black people	4	-
White people	4	-
Indian people	4	-
**Residence**		
Informal community	4	-
Urban	8	-

*Source*: Balbadhur R. Understanding the dignity experience and exploring the impact of dignity therapy and guided imagery on patients with advanced disease – A South African perspective; 2017

Note: Note there are three participants with dual diagnosis (cancer and HIV).

HIV, human immunodeficiency virus; AIDS, acquired immunodeficiency syndrome; CVA, cerebrovascular accident; CCF, congestive cardiac failure.

### Results of thematic analysis

Four major themes and several sub-themes emerged as listed in Table 3.

**TABLE 3:** Themes and sub-themes: Dignity experience of patients with advanced disease.

Themes	Sub-themes
1. Physical concerns	1.1 Loss of independence
	1.2 Symptom burden
	1.3 Frustration and dissatisfaction with medical care
2. Psychological concerns and coping mechanisms	2.1 Anxiety/fear arising from advancing disease
	2.2 Resilience/overcoming adversity
	2.3 Maintaining autonomy
	2.4 Living 1 day at a time
	2.5 Leaving a legacy
	2.6 Role preservation
3. Family and social concerns	3.1 Basic security
	3.2 Social support
	3.3 Compassionate care and respect
	3.4 Stigma
	3.5 Burden to others
	3.6 Concern for loved ones after death
	3.7 Privacy boundaries
4. Spiritual concerns and coping mechanisms	4.1 Support from personal spiritual practice Affirming intrinsic worth Maintaining hope Acceptance and letting go Gratitude
	4.2 Support from organised religion and places of worship
	4.3 Wanting to die/wanting to live

*Source*: Balbadhur R. Understanding the dignity experience and exploring the impact of dignity therapy and guided imagery on patients with advanced disease – A South African perspective; 2017

Numerous participants’ quotes provided the supporting data for the sub-themes (additional quotes can be requested from the author).

### Theme 1: Physical concerns

The theme discusses how illness affects a person’s dignity. Particularly through loss of independence in physical and cognitive abilities with participants describing:

‘There is no dignity… my soul is being sliced away. It affects me very dramatically. Can’t go to the loo by myself. Got to get a nurse to take me. I can’t walk … not being able to get up in the morning.’ (P1, male, 34 years)

‘The worst thing for me will be if I lose my mind … if I forget what I have been and what I want to do.’ (P2, male, 77 years)

The symptom burden experienced by participants impacted their sense of dignity:

‘I just needed a gun; I just wanted to shoot myself and get over with this, because of the pain and weakness. It was unbearable!’ (P3, female, 31 years)

There was frustration and dissatisfaction with medical care:

‘The hospital didn’t give me good care. Doctors do not record our history properly and the files are confused. Different doctors are treating me all the time. I am not getting my treatment … when I used to mess myself, the nurses were unhappy with me and punish me and then I feel I don’t have dignity.’ (P4, male, 70 years)

### Theme 2: Psychological concerns and coping mechanisms

This theme focuses on the mental and emotional distress that participants experienced due to their advancing disease, which affected their sense of dignity.

The sub-theme of anxiety and fears (fear of dependency due to loss of functional and cognitive capacity, being abandoned by medical professionals, being victimised or abused in their vulnerability and loss of role, loss of vitality and poor body image) is illustrated by some quotes:

‘I’m scared of that, being bedridden. I’ll be dependent on who? I don’t know.’ (P5, male, 67 years)

‘… being abandoned by doctors. They can’t treat me anymore. That would crush me completely!’ (P5, male, 67 years)

‘They loved me when I was healthy … look at my photo [*sobbing*], I feel very bad when I see how strong I was and how I am now.’ (P6, female, 42 years)

This theme also explores the mental strategies, qualities or attitudes that participants used to maintain their dignity.

There was a strong sub-theme of resilience or overcoming adversity with one participant describing:

‘When I was diagnosed it hit me, and then realisation hit me that “look, you have got it now, now you have to try to be strong and fight it!” because if you give up and sit down, it is going to overtake you.’ (P7, male, 63 years)

Maintaining autonomy/control is an important aspect of psychological coping:

‘The doctor wanted to do an operation to insert pins in my knees, I felt a bit shaken, and I was not so confident anymore, so I told him I just need time … I was not ready for surgery, and he was fine with it.’ (P8, female, 31 years)

The concept of living one day at a time was expressed:

‘I carry on … I tell my wife, “Don’t worry, we will take things as it comes”… life must go on … no matter how serious the situation is, keep smiling …’ (P7, male, 63 years)

Participants felt that leaving a legacy is important:

‘I can tell the children about my life and tell them to respect themselves and respect everyone. I taught them how to weave grass baskets.’ (P9, female, 46 years)

Role preservation supported participants’ dignity:

‘The ability to work, currently partially, supports my dignity.’ (P5, male, 67 years)

### Theme 3: Family and social concerns

This theme (composed of seven sub-themes) pertains to basic security and the social dynamics within family or healthcare systems that shape an individual’s feeling of dignity.

Participants described concerns about basic security:

‘I was working and now I can’t, I am sick … [*teary*]. I feel very sad because I can’t support my children now … yet I am the breadwinner, and this affects my dignity as my children are hungry.’ (P9, female, 46 years)

Social support is important as one participant explained:

‘Together with the wife, we support and uplift each other, and the children are very supportive.’ (P7, male, 63 years)

Compassionate care and respect were valued:

‘The nurses spoke to me and handled it very professionally. The first time I passed a stool, I was horrified, and they said, “No, these are things you can’t help, it’s natural” … and so I sat and thought about it … I have excellent doctors who listen to what I have to say, and they educate us.’ (P8, female, 31 years)

Lack of respect was also noted:

‘They speak roughly … I don’t get respect from the doctors at the hospital. The way they treat me, they make me feel like crying.’ (P4, male, 70 years)

One of the participants spoke of the impact of stigma:

‘When I touch food, people will not eat it or use the plate or cup that I am using or share the toilet with me. I disclosed to them that I have HIV and cancer and TB … they don’t accept me.’ (P6, female, 42 years)

Participants were concerned about being a burden to others:

‘Sometimes I can’t bathe myself and the neighbours come to bathe me and then I feel life is not worth living …’ (P6, female, 42 years)

Participants expressed concern for loved ones after death:

‘Shame [*crying*] It’s not fair on my son! I am worried about my wife and child, what life am I leaving them?’ (P1, male, 34 years)

There were concerns about privacy boundaries:

‘That was the first time in my life I wore a male nappy. Oh my God! When I was lying in bed and I had to call the sister to change me, that was the worst thing in my life.’ (P10, male, 84 years)

### Theme 4: Spiritual concerns and coping mechanisms

This theme refers to the existential challenges and personal spiritual awareness, attitudes and actions that patients practise to optimise their dignity.

Participants experienced support from personal spiritual practices. Affirming intrinsic worth was illustrated by one participant describing that:

‘I think dignity is something inside you and not something that people can give and take away from you. If you have pride in yourself, then nobody can take that out of you … come the worst situation. You always think of yourself as somebody, not a nothing!’ (P7, male, 63 years)

Maintaining hope was seen as important:

‘I never ever reached that point where I said, “it’s going to end forever,” they gave me till December to live … even then, I didn’t believe them. This inner knowing.’ (P3, female, 31 years)

Acceptance and letting go were described as a learned approach:

‘Now I have taken a more spiritual turn in life; I have been handing over to nurses mentally. I have learnt to let go … and not be so controlling … I say, “Get over it immediately and don’t stress about it.” Through meditation, I just let go. You have got to choose your battles.’ (P8, female, 31 years)

Feeling gratitude was also described:

‘There are people worse off than me so I must thank God we have bread to eat. People are begging. Looking at the bigger picture, our prayer should be for them.’ (P7, male, 63 years)

Participants received support from organised religion and places of worship:

‘I know no one can help me, only God can help me. I pray and God gives me strength. … when I go to church, the word of God boosts my mind, people give me good support and words for counselling, I feel well, and the mind is better.’ (P11, female, 50 years)

The swing from wanting to die to wanting to live was also evident with one participant explaining that:

‘On Dr House, I heard, “You can’t die with dignity, but you have to live with dignity, a dignified life.” And that is when that whole idea went out of my head and suicide is not an option for me. For me when I am at that point, I feel like I am at the bottom and the only way to go is up … It’s 99% of your attitude and positivity and the vibes that you give out … if you give positivity, you attract positivity, so I try and just … stay calm.’ (P8, female, 31 years)

## Discussion

The study population comprised a diverse mix of age, gender, disease and socioeconomic and cultural factors, enhancing the generalisability^21^ of our results. This study may sensitise HCPs, family and society to the difficulties and vulnerabilities experienced by diverse patients and the impact their attitudes and behaviours have on patients’ dignity.

### Acceptability and relevance of the study in a South African setting

The findings indicated that participants were willing to share their insights on dignity, including the factors that enhance or undermine it. This study appeared to be therapeutic for participants who stated that they felt heard and understood by their HCPs after the interview. IsiZulu-speaking participants understood the term ‘dignity’ with additional explanation using concepts of self-worth, self-esteem and self-confidence. Participants described dignity as deep-seated and an internal state of mind, such as pride, respect, esteem and self-worth.^1^

The experience of loss of physical capacity and symptom burden was particularly hard to accept in participants who had a strong sense of independence. The lack of basic security, such as food, shelter, transportation, money and medical assistance, significantly impacted the dignity of those in lower socioeconomic groups. All participants noted the influence of family, community and HCPs on their experience of extrinsic dignity. Respect and compassionate care were important, and participants also commented on the respect and love they received from doctors and nurses.

Participants described uplifting attitudes, how they viewed the situation and spiritual practices that gave their life meaning and enhanced their sense of dignity even though they were dealing with anxieties about their reality and impending death. Use of psychological and spiritual coping mechanisms was described in most interviews, illustrating a profound dependence on spirituality amongst South Africans as described below.

### Model of influences on the total dignity experience of a patient with advanced disease

**FIGURE 2:**
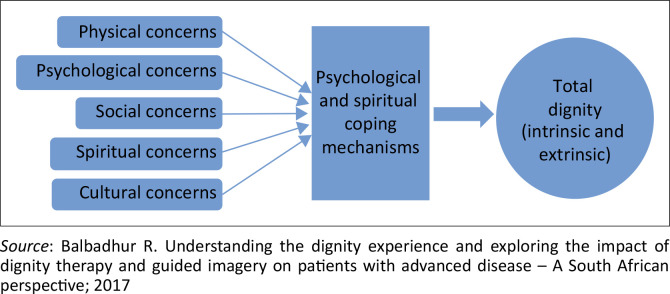
Revised model of total dignity – A South African perspective.

Building on Chochinov’s Dignity model, the theoretical framework for this research study, additional factors were identified as impacting the dignity experience. Physical, psychosocial, spiritual and cultural concerns diminish a person’s sense of dignity. Conversely, respect and caring from others and personal positive psychological and spiritual coping mechanisms enhance the sense of dignity. This study provided unique insight into the impact of culture on total dignity; see a revised dignity model (Figure 2).^1^

The findings suggest that individuals with strong psycho-spiritual coping mechanisms tend to exhibit greater resilience in preserving their dignity when faced with bio-psycho-social-cultural-spiritual challenges. Nonetheless, in cases where these challenges are particularly intense, they may surpass the effectiveness of these coping strategies, leading to potential negative impacts on dignity. Thus, two related approaches are proposed to improve dignity experience in people who are dying. Firstly, total care of the patient is paramount to address bio-psycho-social-cultural-spiritual concerns. Secondly, it is important to identify and support individual psycho-spiritual coping mechanisms.

### Four domains (bio-psycho-social-spiritual) influence the total dignity experience

#### Physical domain

*Illness-related factors*: For highly independent individuals who based their worth on their physical or cognitive capacity to be productive and in control, this study affirmed previous studies illustrating the importance of a level of independence in retaining extrinsic dignity.^7,8,9^ A participant with loss of functioning capacity described his experience as ‘disgusting’ and ‘the worst thing in the world!’ reflecting deep emotional distress. His fastidious inflexible personality hindered acceptance of his illness until death, illustrating how personality traits affect extrinsic dignity and support Chochinov’s claim that ‘who we are’ influences ‘how we die’.^22^ Incontinence and dependency for ablutions caused significant distress, leading some to wish for death, as noted in prior studies.^8,9^ Those unable to accept bodily changes experienced severe suffering, confirming Chochinov’s view that threats to personal identity disturb body, mind and soul.^13^

The burden of symptoms, particularly pain, was severe and caused significant suffering, leading to thoughts of wanting to die. In this study, a determined and resilient individual expressed a desire to die because of untreated pain, which was worsened by psychological distress stemming from a loss of functionality and role. With good pain control and dignity-enhancing interventions, his wish to die was allayed and his suffering lessened, and his life felt more meaningful and purposeful.

*Health system factors*: In South Africa, the overburdened public healthcare system^23,24^ results in brief consultations, long waits for diagnostic tests and specialist appointments and a lack of continuity of care from HCPs, often delaying diagnoses. Participants in this study expressed frustration with poor information sharing from HCPs, feeling that their needs were overlooked because of time pressure. Many felt disrespected and unsupported in their vulnerable states. One participant from a private facility recounted feeling undermined when his physician publicly mocked and dismissed his pain, leaving it untreated.

Chochinov emphasises that HCPs must be mindful of their attitudes and behaviours to provide dignity-conserving care.^13^ Recognising patients as individuals with unique challenges allows their intrinsic dignity to be affirmed. Transitioning from disease-oriented to person-centred care is a vital attitudinal shift in the therapeutic relationship, rooted in the connection between the HCP’s affirmation and the patient’s self-perception.^11^

In an overburdened healthcare system, both patients and HCPs face challenges. Sulmasy notes that treating others without proper esteem is undignified,^25^ highlighting the importance of dignity-conserving care for patients, families and providers. Chochinov adds that caregivers gain dignity from their actions, enhancing their ability to support those nearing death.^11^ Health care providers need ongoing training in compassionate listening, communication, and understanding patients’ holistic distress sources. Additionally, recognising and addressing burnout amongst HCPs is essential,^26,27^ as they require support to thrive in high-stress environments.

#### Psychological domain

In keeping with international studies,^7,8,9^ participants expressed numerous anxieties and fears of loss of functioning and cognitive capacity and their ensuing dependency. Fear of loss of role and a sense of being a burden on others impacted their perception of their value and worth as individuals.^22,28,29^

A participant experienced severe psychological distress because of her loss of vitality and weight. Metz described the significance of vitality and community in African conceptions of dignity,^30^ whereby vitality is an inherent life force, representing a valuable spiritual and invisible energy within all physical entities. This vital force can be compromised by factors such as illness, suffering, fatigue and depression,^30^ underscoring its essential role in the maintenance of dignity.

Some participants in this study confirmed that the concept of body image affects extrinsic dignity, in keeping with other studies.^8,31^ However, two younger participants with good coping skills and social support spoke of the fact that who they are as a person, rather than changes in their bodies, is an important consideration. This illustrates the benefits of affirming intrinsic dignity.

Participants reported feeling significant anxiety stemming from a fear of abandonment by HCPs. Additionally, they experienced distress from being victimised or abused, as well as feeling disrespected by family and community members during their vulnerable times – this included having their property, possessions, money and choice of residence taken away. Some of these fears may be realistic, whilst others may be anticipated, as noted by De Lima.^32^ Regardless, these fears are real for the patients and should be acknowledged and discussed.

*Psychological coping mechanisms*: There were many dignity-conserving strategies employed by participants to preserve their experience of dignity in disease and dying, as also described by Chochinov et al.^6^ It was observed in this study that those who had more challenging lives and greater assaults to their dignity over a prolonged period even with loss of functioning capacity, had greater resilience in overcoming adversity. Autonomy in the home and medical care improved participants’ sense of control and dignity. Participants spoke of living 1 day at a time as this reduced their anxiety about what the future would bring. Leaving a legacy for the family brought comfort and meaning to their lives. Dignity is enhanced through preservation of a person’s role even if this was altered to allow for reduced capacity. Promoting these practices in consultations can contribute to improving patients’ dignity.

#### Social domain

Social issues had a great impact on participants and resulted in considerable distress.

*Basic needs*: In this study, unmet basic needs were a governing theme in the lower socioeconomic group. The basic needs for food, shelter, money and medical assistance, including access to transportation, medication and continuity of care, were scarce and threatened by illness. Physiological needs and safety needs together make up basic needs. If these basic needs are unmet, patients may be unable to progress and meet their social, esteem and self-actualisation needs.^33^ This affects how patients perceive their value and worth as individuals.

In many parts of South Africa, people live in overcrowded conditions or makeshift homes that do not support optimal care. Many families rely on social grants for pensioners or disabled individuals, which are insufficient to meet their needs.^34^ Children become sources of physical and financial support (the intention of childhood grants is to support children, yet these are misused). Patients cannot keep their appointments if they lack transport funds. A wheelchair-bound participant spent nearly a month’s grant on privately arranged transport to and from the hospital, leaving no funds for a subsequent visit that month.^1^ This delays diagnosis and treatment and worsens feelings of suffering as patients feel undervalued. Occasionally, medicines are unavailable in the public healthcare sector,^1^ leaving symptoms untreated, causing suffering. Without unmet basic needs, life seems hopeless and has no meaning or purpose.

*Community as an African conception of dignity*: Community plays a crucial role in supporting personal dignity within an African context.^14,17,30^ The disabling effect of lack of social support and stigma on human dignity amongst the black isiZulu-speaking participants living in informal communities caused immense psychological distress. This distress was illustrated by one participant who had been rejected and disrespected by his family and community and cried throughout his interview. This experience of rejection and isolation was so severe that some participants expressed a wish to die.

Equally, this study identified the role of strong support structures in boosting dignity. Women from all cultural backgrounds described that being alive and supporting their children provided meaning and was a source of resilience.

Highly independent individuals who opted for an isolated lifestyle often faced challenges because of a lack of social support and yet were hesitant to reach out to neighbours or hire assistance. Numerous studies around the world demonstrate the advantages of fostering compassionate community networks that offer care for those with advanced illnesses and support for the dying.^35,36^ This study highlighted the role of hospices and faith-based communities in providing such care.

*Compassionate care and respect from family, community and HCPs*: Participants expressed their appreciation for the compassionate care provided by family and respectful attitude, behaviour and valuable dialogue and communication skills of HCPs, echoing Chochinov’s ABCD of Dignity-Conserving Care described earlier.^13^ The hospice staff offered compassionate nursing support, dietary guidance, a therapeutic presence and assistive devices. Experiencing disrespect was a source of psycho-existential pain and negatively impacted one’s physical health.

*Stigma*: When reviewing work on dignity in end-of-life care in the international literature, stigma was not noted. However, stigma towards people living with HIV was still a concern for participants in this study. In addition, participants from Indian communities isolated themselves from society to keep their diagnosis from others in fear of stigma. However, it was noted that stigma of cancer has lessened as cancer becomes more prevalent.

*Burden to others*: A common concern amongst participants who reported psychosocial distress, such as depression and hopelessness, was the fear of being a burden on others. This is also reported in international studies.^8,22,28,29^ Chochinov et al.^22^ describe that individuals who believe their life lacks value, meaning and purpose may project these feelings onto others, perceiving themselves as a burden. Encouraging open dialogue amongst individuals and their families regarding feelings of being a burden can significantly mitigate feelings of isolation. Family members often desire to provide support. Engaging in these conversations can foster a deeper understanding of each other’s experiences, needs and the desire for mutual assistance, promoting a more supportive caring environment. As Giles Fraser said:

I do want to be a burden on my loved ones just as I want them to be a burden on me – it’s called looking after each other. For it is when we are this vulnerable, that we have little choice but to allow ourselves to be loved and looked after.^37^

The experience of loss of privacy during care in advanced illness can result in people feeling ashamed or humiliated.^38,39^ Providing privacy and normalising the care processes when assisting with ablutions and ensuring respectful covering of patients are key considerations in providing dignity-conserving care.

#### Spiritual domain

This study highlights the significant role of spirituality amongst South African people in preserving dignity, which fosters resilience in extreme adversity. As compared to other studies,^6,8,38^ key themes that emerged were seeking spiritual support from personal spiritual practices, or from organised religion and places of worship, and faith and trust in God and prayer. Those who had a strong spiritual or religious affiliation seemed to have stronger coping mechanisms as discussed by Cole.^40^ This implies that patients with a belief system should be encouraged to engage in practices that promote strength, peace of mind and closure.

In this study, participants also employed numerous personal spiritual practices to find meaning in their challenging life experiences:

*Affirming intrinsic worth*: Participants acknowledged and affirmed an intrinsic worth that is ‘deep inside’ and that could not be taken away from them. This echoes the Universal Declaration of Human Rights Article 1 of 1948.^41^ A participant described that freedom of choice in how she responded to challenges was important. She endeavoured not to allow other people or difficult situations to decide her responses. Individuals subjected to severe physical and social adversity asserted that their dignity could not be diminished by anyone. These South African participants showed a deep awareness of human dignity and a recognition that dignity belongs to each individual who possesses unique inherent, unconditional intrinsic worth.

*Maintaining hope*: In this study, participants expressed a hopefulness that defied prognosis and demonstrated confidence in themselves despite their challenges. They also felt they could uplift those who were in worse circumstances than themselves. As described in other studies,^6,10^ maintaining hope supports dignity. This hope emerged when they found meaning in their challenges, as described by Chochinov.^6^ Hope demonstrated the ability to ignite a strength and perseverance of spirit that defied physical norms.

*Acceptance and letting go*: Acceptance and letting go is a concept that is associated with a ‘good death’.^6,10,38^ Within this study, participants expressed acceptance and letting go to be associated with a strong religious or spiritual base, and a practice that matures with age, patience and time along the disease trajectory. Letting go of concerns over which participants no longer had control, was associated with a feeling of liberation. Furthermore, a study conducted by Cole found that letting go can be advantageous for managing situations that are beyond one’s personal control.

*Gratitude*: A theme that has not been addressed in international studies is the role of gratitude and appreciation in enabling participants to uphold their dignity. Specifically, the experience of gratitude facilitated a sense of transcending their circumstances and contributed to the search for meaning in their lives.

### Limitations of the study

The small sample size is a potential limitation, even with a balanced representation of ages, genders, races and advanced diseases. The accuracy of the translated data depended on the isiZulu-speaking caregiver.

### Recommendations

*Physical concerns* highlight the necessity for regular assessments and attentive symptom management through holistic interdisciplinary approaches, along with precise communication and information sharing. As Krakauer et al. describe education of primary HCPs in the use of analgesia and non-pharmacological methods to treat pain will improve the dignity of patients debilitated by pain.^42^

The distress from loss of vitality and cachexia can be supported with exercise and careful clinical management of gastrointestinal diseases, fatigue and depression. When cachexia reaches an irreversible stage, it is essential to provide patients with appropriate counselling and support. They must understand that, despite their physical decline, their intrinsic spirit and life force remain intact.

*Psychological* distress needs to be supported by HCPs who can offer compassionate listening to acknowledge and understand patients’ anxieties and offer counselling. Often simply being present to listen to their fears and anxieties is therapeutic for the patient.

Patients should be encouraged to employ *psychological coping strategies* to navigate adversity and actively engage in decision-making related to both medical and personal issues. Participation in routine activities, momentary distractions and customary roles is vital for their well-being. Healthcare professionals must recognise and demonstrate interest in the aspects of life that patients hold in the highest regard.

Addressing basic security needs requires the engagement of *social workers* and systemic advocacy. Social workers can expand social support networks by integrating hospice and home-based care. Promoting awareness campaigns can destigmatise HIV and life-threatening illnesses, fostering societal understanding of patient vulnerability and the need for compassionate care. Promoting compassionate community networks nationally is important. Encouraging family discussions about patients’ concerns – especially regarding fears of burden – can facilitate practical support. Conversations regarding advanced care directives, wills and funeral planning are also important. Additionally, maintaining patient privacy and obtaining permission before examinations is vital.

Recognising the underlying distress that may accompany expressions of a desire to die is essential in providing appropriate comfort care. It is crucial to connect patients with *spiritual* organisations and leaders, fostering opportunities for their engagement in culturally and spiritually meaningful practices, thereby enhancing their overall sense of well-being.

A critically important intervention is to encourage and support people to develop better *spiritual coping mechanisms* focusing on their intrinsic worth. See the patient as an individual worthy of respect, honour and esteem. The affirmation of a person’s non-physical identity and intrinsic worth is particularly important as their physical health declines. As Brennan stated, patients need to be reminded that nothing can take away their spirit, *who they are*, not even serious illness.^3^ Addressing suffering because of the existential pain of a loss of identity and role, can reduce total pain.

A larger study can be done to understand the dignity experience amongst the heterogenous cultural landscape in South Africa, different diseases and along different disease trajectories.

Experiential workshops integrated into undergraduate training can help HCPs develop essential qualities like kindness, compassion and respect for individuals. Implementing *supportive programmes for HCPs* is vital, as their well-being directly enhances their ability to provide compassionate care, ultimately improving patient outcomes and fostering a more humane healthcare environment.

## Conclusion

Dignity is an intrinsic unconditional quality of human worth. This study introduces a revised model indicating that dignity for patients with advanced illness is influenced by bio-psycho-social, cultural and spiritual concerns. In South Africa, individuals face challenges that shape their dignity experiences based on their personality and socioeconomic-cultural backgrounds, especially amongst lower socioeconomic groups, where unmet basic needs, inadequate social support and a lack of compassionate care from family, community and HCPs significantly impact their dignity in advanced illness.

This study illustrates the resilience of South Africans, who employ various psycho-spiritual coping strategies despite enduring assaults on their dignity. They maintain a belief in the inherent worth of each individual, which remains intact regardless of external challenges and cannot be taken away by people or illness. They recognise their importance as individuals who have rights, dignity and personal worth.

It is essential that primary HCPs offer kindness, compassion and unconditional respect that acknowledges and honours each individual’s inherent value beyond their illness, focusing on *who they are*, rather than their condition. Patients need to be seen as living beings, not disease entities that need fixing. Patients should be: (1) reminded of their unique untouchable worth; (2) encouraged to seek and trust in spiritual support; and (3) supported to find meaning and purpose in their last days. This study shows that those who embraced these principles maintained their dignity even when faced with significant hardships, valuing their imperishable worth.
